# Clinical practice recommendations for the diagnosis and management of X-linked hypophosphataemia

**DOI:** 10.1038/s41581-019-0152-5

**Published:** 2019-05-08

**Authors:** Dieter Haffner, Francesco Emma, Deborah M. Eastwood, Martin Biosse Duplan, Justine Bacchetta, Dirk Schnabel, Philippe Wicart, Detlef Bockenhauer, Fernando Santos, Elena Levtchenko, Pol Harvengt, Martha Kirchhoff, Federico Di Rocco, Catherine Chaussain, Maria Louisa Brandi, Lars Savendahl, Karine Briot, Peter Kamenicky, Lars Rejnmark, Agnès Linglart

**Affiliations:** 1 Department of Pediatric Kidney, Liver and Metabolic Diseases, Hannover Medical School, Hannover, Germany; 2 Center for Congenital Kidney Diseases, Center for Rare Diseases, Hannover Medical School, Hannover, Germany; 3 Department of Pediatric Subspecialties, Division of Nephrology, Children’s Hospital Bambino Gesù – IRCCS, Rome, Italy; 4 Department of Orthopaedics, Great Ormond St Hospital for Children, Orthopaedics, London, UK; 5 The Catterall Unit Royal National Orthopaedic Hospital NHS Trust, Stanmore, UK; 6 Dental School, Université Paris Descartes Sorbonne Paris Cité, Montrouge, France; 7 APHP, Department of Odontology, Bretonneau Hospital, Paris, France; 8 APHP, Reference Center for Rare Diseases of Calcium and Phosphate Metabolism, and Filière OSCAR, Paris, France; 9 Department of Pediatric Nephrology, Rheumatology and Dermatology, University Children’s Hospital, Lyon, France; 10 Center for Chronic Sick Children, Pediatric Endocrinology, Charitè, University Medicine, Berlin, Germany; 11 APHP, Department of Pediatric Orthopedic Surgery, Necker – Enfants Malades University Hospital, Paris, France; 12 Paris Descartes University, Paris, France; 13 University College London, Centre for Nephrology and Great Ormond Street Hospital for Children NHS Foundation Trust, London, UK; 14 Hospital Universitario Central de Asturias (HUCA), University of Oviedo, Oviedo, Spain; 15 Department of Pediatric Nephrology and Development and Regeneration, University Hospitals Leuven, University of Leuven, Leuven, Belgium; 16 RVRH-XLH, French Patient Association for XLH, Suresnes, France; 17 Phosphatdiabetes e.V., German Patient Association for XLH, Lippstadt, Germany; 18 Pediatric Neurosurgery, Hôpital Femme Mère Enfant, Centre de Référence Craniosténoses, Université de Lyon, Lyon, France; 19 Metabolic Bone Diseases Unit, Department of Surgery and Translational Medicine, University of Florence, Florence, Italy; 20 Pediatric Endocrinology Unit, Karolinska University Hospital, Department of Women’s and Children’s Health, Karolinska Institutet, Stockholm, Sweden; 21 APHP, Department of Rheumatology, Cochin Hospital, Paris, France; 22 INSERM UMR-1153, Paris, France; 23 APHP, Department of Endocrinology and Reproductive Diseases, Bicêtre Paris-Sud Hospital, Paris, France; 24 INSERM U1185, Bicêtre Paris-Sud, Paris-Sud - Paris Saclay University, Le Kremlin-Bicêtre, France; 25 Department of Endocrinology and Internal Medicine, Aarhus University Hospital, Aarhus, Denmark; 26 APHP, Platform of Expertise of Paris-Sud for Rare Diseases and Filière OSCAR, Bicêtre Paris-Sud Hospital (HUPS), Le Kremlin-Bicêtre, France; 27 APHP, Endocrinology and Diabetes for Children, Bicêtre Paris-Sud Hospital, Le Kremlin-Bicêtre, France

**Keywords:** Phosphorus metabolism disorders, Bone, Therapeutics, Clinical genetics

## Abstract

X-linked hypophosphataemia (XLH) is the most common cause of inherited phosphate wasting and is associated with severe complications such as rickets, lower limb deformities, pain, poor mineralization of the teeth and disproportionate short stature in children as well as hyperparathyroidism, osteomalacia, enthesopathies, osteoarthritis and pseudofractures in adults. The characteristics and severity of XLH vary between patients. Because of its rarity, the diagnosis and specific treatment of XLH are frequently delayed, which has a detrimental effect on patient outcomes. In this Evidence-Based Guideline, we recommend that the diagnosis of XLH is based on signs of rickets and/or osteomalacia in association with hypophosphataemia and renal phosphate wasting in the absence of vitamin D or calcium deficiency. Whenever possible, the diagnosis should be confirmed by molecular genetic analysis or measurement of levels of fibroblast growth factor 23 (FGF23) before treatment. Owing to the multisystemic nature of the disease, patients should be seen regularly by multidisciplinary teams organized by a metabolic bone disease expert. In this article, we summarize the current evidence and provide recommendations on features of the disease, including new treatment modalities, to improve knowledge and provide guidance for diagnosis and multidisciplinary care.

## Abstract (web summary)

In this Evidence-Based Guideline on X-linked hypophosphataemia, the authors identify the criteria for diagnosis of this disease, provide guidance for medical and surgical treatment and explain the challenges of follow-up.

## Introduction

X-linked hypophosphataemia (XLH) is an X-linked dominant disorder caused by mutations in *PHEX* (located at Xp22.1), which encodes a cell-surface-bound protein-cleavage enzyme (phosphate-regulating neutral endopeptidase PHEX), predominantly expressed in osteoblasts, osteocytes and teeth (odontoblasts and cementoblasts). XLH is the most common cause of inherited phosphate wasting, with an incidence of 3.9 per 100,000 live births and a prevalence ranging from 1.7 per 100,000 children to 4.8 per 100,000 persons (children and adults)^1–3^. Although the pathogenesis of XLH is not fully understood, animal studies indicate that loss of *Phex* function results in enhanced secretion of the phosphaturic hormone fibroblast growth factor 23 (FGF23), with osteocytes being the primary source of FGF23 production^4^. These effects explain most of the characteristic features of the disease, including renal phosphate wasting with consequent hypophosphataemia, diminished synthesis of active vitamin D (1,25(OH)_2_ vitamin D), rickets, osteomalacia, odontomalacia and disproportionate short stature^4–6^. Patients usually develop clinical symptoms during the first or second year of life. Early treatment with oral phosphate supplementation and active vitamin D heals rickets, limits dental abscess formation and prevents progressive growth failure, but in a substantial proportion of patients treatment is unsuccessful and/or associated with adverse effects (for example, hyperparathyroidism and nephrocalcinosis)^7,8^. Up to two-thirds of children with XLH require lower limb surgery^9–12^. Conventional therapy further stimulates FGF23 levels and thereby renal phosphate wasting, resulting in a vicious circle, which might limit its efficacy^6,13–15^. Adult patients with XLH are at risk of complications such as early osteoarthritis, enthesopathies, spinal stenosis, pseudofractures and hearing loss, which might limit quality of life^16–18^. In 2018, burosumab, a fully human monoclonal IgG1 antibody neutralizing FGF23, was approved by health authorities for the treatment of patients with XLH in the European Union and the USA on the basis of encouraging clinical trial results^19–25^. Treatment with phosphate and/or active vitamin D does not decrease or prevent the development of osteoarthritis or enthesopathies^17,26^. Currently, evidence that treatment with burosumab ameliorates these complications is lacking.

Owing to the rarity of XLH and the diversity of clinical manifestations, diagnosis is often delayed and treatment can be challenging. To date, except for brief guidance provided by two expert groups, no evidence-based, systematically developed recommendations for the diagnosis and management of XLH exist^7,8^. Therefore, an initiative to develop recommendations for the diagnosis and management of patients with XLH was conducted from June 2017 to December 2018, and the recommendations are provided in this Evidence-Based Guideline. These clinical practice recommendations are endorsed by the European Society for Paediatric Nephrology (ESPN), the European Society for Paediatric Endocrinology (ESPE), the European Society of Endocrinology (ESE), the European Reference Network on Rare Endocrine Conditions (Endo-ERN), the European Reference Network on Rare Bone Disorders (BOND), the International Osteoporosis Foundation (IOF) Skeletal Rare Diseases Working Group, the European Calcified Tissue Society (ECTS), the European Paediatric Orthopaedic Society (EPOS) study group on Metabolic and Genetic Bone Disorders, the European Society of Craniofacial Surgery, the European Society for Paediatric Neurosurgery and the European Federation of Periodontology (EFP) and will be revised and endorsed periodically.

## Material and methods

### Overview of the guideline project

We followed the RIGHT (Reporting Items for Practice Guidelines in Healthcare) Statement for Practice Guidelines^27^. Two groups were assembled: a core leadership group and a voting panel. The core group comprised specialists from (paediatric) endocrinology (D.S., M.L.B., L.S., P.K., L.R. and A.L.), paediatric nephrology (D.H., F.E., J.B., D.B., F.S. and E.L.), paediatric orthopaedic surgery (D.E. and P.W.), rheumatology (K.B.), dentistry (M.B.D. and C.C.), neurosurgery (F.D.R.) and XLH patient organization representatives (P.H. and M.K.). The voting group included 41 members with expertise in paediatric and adult XLH, including members of the supporting societies and networks. Voting group members were asked by use of an e-questionnaire to provide a level of agreement on a five-point scale (strongly disagree, disagree, neither agree/disagree, agree or strongly agree) (Delphi method). Failing a 70% level of consensus, recommendations were modified after discussion in the core group and reviewed again by the voting panel until a consensus level of at least 70% was achieved.

### Developing the PICO questions

We developed PICO (patient (or population) covered, intervention, comparator and outcomes) questions^28^. These PICO elements were arranged into the questions to be addressed in the literature searches. Each PICO question then formed the basis for a recommendation.

The population covered included children and adults with XLH. Recommendations have been developed on the basis of available studies investigating the clinical phenotype and management of XLH. For these recommendations, treatment benefits were evaluated using the no treatment option or patient status at baseline (that is, before therapy) as the comparator. With regard to outcomes, we provide recommendations for diagnosis, follow-up and treatment with respect to bone disease, growth, treatment-associated or disease-associated complications and comorbidities.

### Literature search

The PubMed database was searched until 26 June 2018; all articles and reports were considered, including prospective randomized controlled trials, uncontrolled or observational studies, registries, summaries and case reports, restricted to human studies in English. The following key MeSH terms were used to identify suitable studies: “X-linked hypophosphatemia”, “X-linked hypophosphatemic rickets”, “hypophosphatemic rickets”, “familial hypophosphatemic rickets”, “PHEX” and “osteomalacia”. The search retrieved 8,903 results and 196 articles were referenced here.

### Grading system

**Fig. 1:**
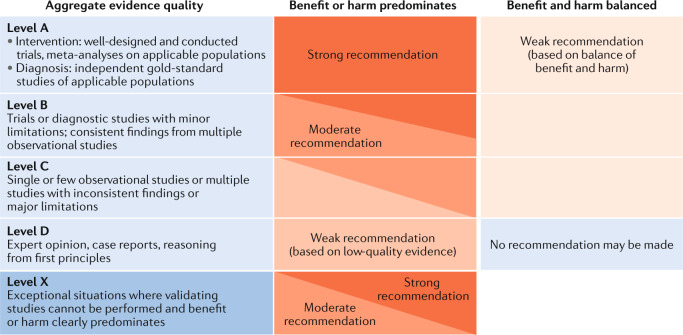
Determining levels of evidence and strength of recommendations (American Academy of Pediatrics grading matrix). Reproduced with permission from ref.^30^: *Pediatrics* 140, e20171904 Copyright © 2017 by the AAP.

We followed the grading system from the American Academy of Pediatrics to develop the recommendations (Fig. 1; refs^29,30^). The quality of evidence is graded high (A), moderate (B), low (C), very low (D) or not applicable (X). The latter refers to exceptional situations in which validating studies cannot be performed and benefit or harm clearly predominates. This letter was used to grade contraindications for burosumab and safety recommendations for cinacalcet. The strength of a recommendation is graded strong, moderate, weak or discretionary (when no recommendation can be made).

## Diagnosis

### General approach

The diagnosis of XLH is based on the association of clinical, radiological and biochemical findings (Box 1). In patients with a negative family history (approximately one-third of reported patients^31,32^), mutational analysis of the *PHEX* gene is recommended, which can provide negative or positive confirmation in ~70–90% of cases^31,33–43^. Specific molecular genetic defects such as large deletions, deletions removing pseudo-exons of *PHEX* or mosaicism can be difficult to identify^44,45^. With atypical clinical presentations and/or negative genetic analyses, further biochemical, molecular genetic and radiological work-up is recommended. The identification of a pathogenic *PHEX* variant in an index case requires genetic counselling and enables screening of relatives at risk.

Box 1 Recommendations for diagnosis

- In children, a diagnosis of X-linked hypophosphataemia (XLH) should be considered in the presence of clinical and/or radiological signs of rickets, impaired growth velocity and serum levels of phosphate below the age-related reference range associated with renal phosphate wasting and in the absence of vitamin D or calcium deficiency (grade B, moderate recommendation)
- In adults, the diagnosis of XLH should be considered in the presence or history of lower limb deformities, and/or clinical and/or radiological signs of osteomalacia (including pseudofractures, early osteoarthritis and enthesopathies) in the context of serum levels of phosphate below the age-related reference range associated with renal phosphate wasting (grade B, moderate recommendation)
- We recommend that any first-generation family member of a patient with XLH should be investigated for XLH (grade D, weak recommendation); sons of males affected by XLH are not at risk
- We recommend the following initial diagnostic work-up (grade B, moderate recommendation):
  - A detailed clinical evaluation, including evidence of rickets, growth failure, dental abnormalities and signs of craniosynostosis and/or intracranial hypertension
  - A radiological evaluation to diagnose and grade rickets and osteomalacic lesions
  - Biochemical tests, including serum levels of phosphate, calcium, alkaline phosphatase, parathyroid hormone, 25(OH) vitamin D, 1,25(OH)_2_ vitamin D and creatinine, and urinary levels of calcium, phosphate and creatinine by use of a spot urine test for calculation of the tubular maximum reabsorption of phosphate per glomerular filtration rate (TmP/GFR) and urinary calcium:creatinine ratio

- We recommend that non-selective renal tubular phosphate wasting (which suggests renal Fanconi syndrome) should be excluded by looking for abnormal bicarbonate, amino acid, glucose and/or uric acid losses in urines and low molecular mass proteinuria (grade B, moderate recommendation)
- We recommend confirming the clinical diagnosis of XLH by genetic analysis of the *PHEX* gene in children and adults if feasible (grade B, moderate recommendation)
- If genetic analysis is not available, elevated plasma levels of intact fibroblast growth factor 23 (FGF23) and/or a positive family history for XLH support the diagnosis (grade C, moderate recommendation)
- We recommend other causes of hereditary or acquired hypophosphataemia be considered if analysis of the *PHEX* gene yield a negative result for XLH (grade B, moderate recommendation)
- We recommend genetic counselling be offered to patients with XLH, especially at the transition from child to adult care and to families planning pregnancies (grade C, moderate recommendation)
- Methods for detecting *PHEX* mutations can be applied to preimplantation genetic diagnosis or prenatal diagnosis. However, recommendations should be adapted to country-specific ethical and legal standards and communicated using appropriate genetic counselling (grade D, weak recommendation)
- We recommend a further work-up after diagnosis including investigations aimed at diagnosing the presence and severity of common and rare complications of the disease summarized in Table 1 (grade C, moderate recommendation)

### Clinical features

In children, the main clinical symptoms of XLH are abnormal gait, lower limb deformity and decreased growth velocity. Dental abscesses are highly prevalent in patients >3 years of age^46,47^. In undiagnosed adults, typical findings of XLH include short stature, osteomalacia, bone pain, osteoarthritis, pseudofractures, stiffness, enthesopathies and poor dental condition including periodontitis (inflammation of the gums)^16–18,48–50^. Osteomalacia-related bone pain needs to be distinguished from osteoarthritis-related bone pain. Rachitic skeletal deformities usually become apparent at the age of 6 months. During the second year of life, patients present with delayed walking, a waddling gait, progressive lower limb deformities (varus deformity or valgus deformity) often combined with a torsional component (intoeing or extoeing), widening of the distal metaphyses at the wrist and ankle level, thickening of the costochondral junctions and reduced growth velocity^51^. Impaired limb growth with relatively preserved trunk growth results in disproportionate short stature^7,8,17,49,52^. Patients might present with an abnormal skull shape — that is, dolichocephaly, characterized by parietal flattening, frontal bossing and widened sutures as the consequence of premature fusion of the parietal and frontal bones^53,54^.

### Radiographic manifestations

**Fig. 2:**
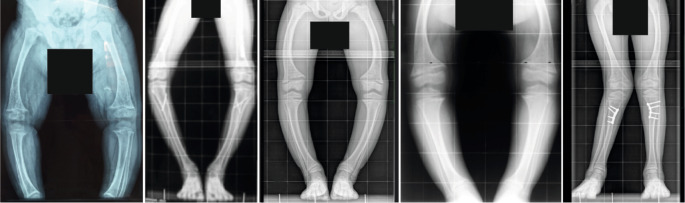
Radiographs of the lower extremities of children affected with X-linked hypophosphataemia. The patients show disproportionate short stature with genu varum (bowed legs). The final panel on the right shows a patient with a windswept deformity (characterized by a valgus deformity in one knee in association with a varus deformity in the other knee). The radiographs reveal severe leg bowing, partial fraying and irregularity of the distal femoral and proximal tibial growth plates. Note the lack of bone resorption features.

Rachitic lesions are characterized by cupped and flared metaphyses and widened and irregular physes (growth plates) of the long bones. In contrast to rickets that is secondary to vitamin D or calcium deficiency, in XLH, cortical bone often appears thickened and features of bone resorption are lacking. These abnormalities preferentially occur at sites of rapid growth (in particular the distal femora, distal tibiae and distal radii) and typically affect costochondral junctions, which leads to the development of the rachitic rosary and Harrison’s groove. Radiography limited to the knees and/or wrists and/or the ankles is usually sufficient to diagnose rickets^8^. Bone deformities primarily involve lower limbs (Fig. 2). Adults might present with different radiographic features from children, including pseudofractures, early osteoarthritis of the spine, hip and knees (osteophytes on joint margins or narrowing of joint cartilage) and/or enthesopathies (such as bone proliferation at the site of ligament attachments or calcification of ligaments)^16,17^. Osteomalacia-related fractures are rarely observed in adults, whereas pseudofractures are frequently observed in adults^16,17,55,56^.

### Biochemical characteristics

The biochemical hallmarks of XLH are hypophosphataemia due to renal phosphate wasting, increased alkaline phosphatase (ALP) levels and elevated intact FGF23 levels^7,8^. A positive family history of XLH, elevated ALP levels, decreased serum phosphate concentrations associated with renal phosphate wasting and/or the identification of a *PHEX* mutation can help to identify affected children within the first weeks of life. Of note, serum levels of phosphate might be in the normal range within the first 3–4 months of life^57,58^. Renal phosphate wasting should be evaluated by calculating the tubular maximum reabsorption of phosphate per glomerular filtration rate (TmP/GFR)^59^ (Table 1). In patients with insufficient phosphate intake, or impaired intestinal absorption (which might be suspected by low urinary levels of phosphate), TmP/GFR can be falsely low until serum levels of phosphate are restored to normal. Although, plasma levels of intact FGF23 are usually elevated, normal levels of FGF23 do not exclude XLH but should be interpreted as inappropriately normal in the setting of hypophosphataemia^60^. FGF23 levels are also influenced by other factors, in particular phosphate intake and vitamin D therapy^2,14,15,60^. Therefore, FGF23 levels are most informative in untreated patients^6^. FGF23 levels are elevated in several other forms of hypophosphataemic rickets (Table 2), and the normal range varies considerably according to the assay used^14,61^. Serum concentrations of calcium are usually in the lower normal range, and urinary calcium is low owing to impaired 1,25(OH)_2_ vitamin D synthesis and consequently decreased intestinal calcium absorption. In contrast to calcipenic rickets, parathyroid hormone (PTH) levels are usually at the upper limit of the normal range or even slightly elevated. Circulating levels of 1,25(OH)_2_ vitamin D are low or inappropriately normal in the setting of hypophosphataemia^6,62–64^.

**Table 1:** Initial evaluation of common and rare complications of XLH

Evaluation	Age of patient		
	<5 years	5–18 years	Adults
***Clinical***			
Growth chart	✓	✓	✓
Signs of rickets and/or leg deformity	✓	✓	✓
Measure IMD and ICD^a^	✓	✓	✓
Head circumference and skull shape	✓	✓	NA
Neurological examination (for consequences of craniosynostosis and spinal stenosis)	✓	✓	✓
Hearing assessment	NA	✓	✓
Dental and oral examination	✓^b^	✓	✓
Musculoskeletal function (gait)^77^	NA	✓	NA
***Biochemistry***			
Blood: calcium, phosphate and creatinine	✓	✓	✓
Spot urine: calcium, phosphate and creatinine^c^	✓	✓	✓
TmP/GFR^d^ (refs^59,193^)	✓	✓	✓
Estimated GFR^194,195^	✓	✓	✓
25(OH) vitamin D	✓	✓	✓
1,25(OH)_2_ vitamin D	✓	✓	✓
PTH	✓	✓	✓
ALP (children) and BAP (adults)	✓	✓	✓
Intact FGF23 (in case of negative family history)	✓	✓	✓
***Imaging***			
Wrist and/or knee and/or ankle radiographs (rickets)	✓	✓	NA
Standardized, well-positioned anterior–posterior standing limb alignment radiograph (using low-dose techniques if possible)^e^	✓	✓	✓
Dental orthopantomogram^f^	NA	✓	✓
Brain MRI^g^	✓	✓	✓
Renal ultrasonography (nephrocalcinosis)	✓	✓	✓

ALP, alkaline phosphatase; BAP, bone alkaline phosphatase; FGF23, fibroblast growth factor 23; GFR, glomerular filtration rate; ICD, intercondylar distance; IMD, intermalleolar distance; NA, not applicable; PTH, parathyroid hormone; TmP/GFR, maximum rate of renal tubular reabsorption of phosphate per glomerular filtration rate; XLH, X-linked hypophosphataemia. ^a^Patient standing with weight on both feet and feet hip-width apart; alternatively, patient lying down. Reference values are given elsewhere^73^. ^b^Starting at 3 years of age. ^c^Upper normal range of calcium:creatinine ratio (mol/mol): 2.2 (<1 year), 1.4 (1–3 years), 1.1 (3–5 years), 0.8 (5–7 years) and 0.7 (>7 years). ^d^Normal range in infants and children (6 months to 6 years): 1.2–2.6 mmol/l; adults: 0.6–1.7 mmol/l (refs^59,193^); a web calculator is found elsewhere^194^. ^e^Low irradiation system (for example, EOS) and check for pseudofractures in adults. ^f^On the basis of clinical needs. ^g^In the presence of a skull morphology in favour of craniosynostosis or clinical signs of increased intracranial pressure (for example, persistent headache or vomiting).

**Table 2:** Characteristics of inherited or acquired causes of phosphopenic rickets in comparison to calcipenic rickets

Disorder (abbreviation; OMIM#)	Gene (location)	Ca	P	ALP	U_Ca_	U_P_	TmP/GFR	FGF23	PTH	25 (OH)D^a^	1,25 (OH)_2_D	Pathogenesis
***Rickets and/or osteomalacia with high PTH levels (calcipenic rickets)***												
Nutritional rickets (vitamin D and/or calcium deficiency)	NA	N, ↓	N, ↓	↑↑↑	↓	Varies	↓	N	↑↑↑	↓↓, N	Varies	Vitamin D deficiency
Vitamin-D-dependent rickets type 1A (VDDR1A; OMIM#264700)	*CYP27B1* (12q14.1)	↓	N, ↓	↑↑↑	↓	Varies	↓	N, ↓	↑↑↑	N	↓	Impaired synthesis of 1,25(OH)_2_D
Vitamin-D-dependent rickets type 1B (VDDR1B; OMIM#600081)	*CYP2R1* (11p15.2)	↓	N, ↓	↑↑↑	↓	Varies	↓	N	↑↑↑	↓↓	Varies	Impaired synthesis of 25(OH)D
Vitamin-D-dependent rickets type 2A (VDDR2A; OMIM#277440)	*VDR* (12q13.11)	↓	N, ↓	↑↑↑	↓	Varies	↓	N, ↓	↑↑↑	N	↑↑	Impaired signalling of the VDR
Vitamin-D-dependent rickets type 2B (VDDR2B; OMIM#164020)	*HNRNPC* (14q11.2)	↓	N, ↓	↑↑↑	↓	Varies	↓	N	↑↑↑	N	↑↑	Impaired signalling of the VDR
Vitamin-D-dependent rickets type 3 (VDDR3; OMIM# pending)	*CYP3A4* (7q21.1)	↓	↓	↑↑↑	↓	Varies	↓	?	↑↑↑	↓	↓	↑ Inactivation of 1,25(OH)_2_D
***Phosphopenic rickets and/or osteomalacia due to dietary phosphate deficiency or impaired bioavailability***												
• Breastfed very-low-birthweight infants	NA	N, ↑	↓	↑, ↑↑	?	↓	N^b^	N, ↓	N	N	N, ↑	Phosphate deficiency
• Use of elemental or hypoallergenic formula diet or parental nutrition												
• Excessive use of phosphate binders												
• Gastrointestinal surgery or disorders												
***Phosphopenic rickets and/or osteomalacia with renal tubular phosphate wasting due to elevated FGF23 levels and/or signalling***												
X-linked hypophosphataemia (XLH; OMIM#307800)	*PHEX* (Xp22.1)	N	↓	↑, ↑↑	↓	↑	↓	↑, N	N, ↑^c^	N	N^d^	↑ FGF23 expression in bone and impaired FGF23 cleavage
Autosomal dominant hypophosphataemic rickets (ADHR; OMIM#193100)	*FGF23* (12p13.3)	N	↓	↑, ↑↑	↓	↑	↓	↑, N	N, ↑^c^	N	N^d^	FGF23 protein resistant to degradation
Autosomal recessive hypophosphataemic rickets 1 (ARHR1; OMIM#241520)	*DMP1* (4q22.1)	N	↓	↑, ↑↑	↓	↑	↓	↑, N	N, ↑^c^	N	N^d^	↑ FGF23 expression in bone
Autosomal recessive hypophosphataemic rickets 2 (ARHR2; OMIM#613312)	ENPP1 (6q23.2)	N	↓	↑, ↑↑	↓	↑	↓	↑, N	N, ↑^c^	N	N^d^	↑ FGF23 expression in bone
Raine syndrome associated (ARHR3; OMIM#259775)	*FAM20C* (7q22.3)	N	↓	↑, ↑↑	?	↑	↓	↑, N	N, ↑^c^	N	N^d^	↑ FGF23 expression in bone
Fibrous dysplasia (FD; OMIM#174800)	*GNAS (*20q13.3)	N, ↓	↓	↑, ↑↑	↓	↑	↓	N, ↑	N, ↑^c^	N	N^d^	↑ FGF23 expression in bone
Tumour-induced osteomalacia (TIO)	NA	N, ↓	↓	↑, ↑↑	↓	↑	↓	N, ↑	N, ↑^c^	N	N^d^	↑ FGF23 expression in tumoural cells
Cutaneous skeletal hypophosphataemia syndrome (SFM; OMIM#163200)	*RAS* (1p13.2)	N, ↓	↓	↑, ↑↑	↓	↑	↓	N, ↑	N, ↑^c^	N	N^d^	Unknown
Osteoglophonic dysplasia (OGD; OMIM#166250)	*FGFR1* (8p11.23)	N	↓	↑, N	N	↑	↓	N	N, ↑^c^	N	N^d^	↑ FGF23 expression in bone
Hypophosphataemic rickets and hyperparathyroidism (OMIM#612089)	*KLOTHO* (13q13.1)	N	↓	↑, ↑↑	↓	↑	↓	↑	↑↑	N	N^d^	Unknown; translocation of the KLOTHO promoter
***Phosphopenic rickets and/or osteomalacia due to primary renal tubular phosphate wasting***												
Hereditary hypophosphataemic rickets with hypercalciuria (HHRH; OMIM#241530)	*SLC34A3* (9q34.3)	N	↓	↑(↑↑)	N, ↑	↑	↓	↓	Low N, ↓	N	↑↑	Loss of function of NaPi2c in the proximal tubule
X-linked recessive hypophosphataemic rickets (OMIM#300554)	*CLCN5* (Xp11.23)	N	↓	↑(↑↑)	N, ↑	↑	↓	Varies	Varies	N	↑	Loss of function of CLCN5 in the proximal tubule
Hypophosphataemia and nephrocalcinosis (NPHLOP1; OMIM#612286) and Fanconi reno-tubular syndrome 2 (FRTS2; OMIM#613388)	*SLC34A1* (5q35.3)	N	↓	↑(↑↑)	↑	↑	↓	↓	Varies	N	↑	Loss of function of NaPi2a in the proximal tubule
Cystinosis (OMIM#219800) and other hereditary forms of Fanconi syndrome	*CTNS* (17p13.2)	N, ↓	↓	↑(↑↑)	N, ↑	↑	N, ↓	N, ↑^e^	N, ↑^e^	N	N^d^	Cysteine accumulation in the proximal tubule
Iatrogenic proximal tubulopathy	NA	N	↓	↑(↑↑)	Varies	↑	↓	↓	Varies	N	↑	Drug toxicity

N, normal; ↑, elevated; ↑↑ or ↑↑↑, very elevated; ↑(↑↑), might range widely; 1,25(OH)_2_D, 1,25-dihydroxyvitamin D; 25(OH)D, cholecalciferol; ALP, alkaline phosphatase; Ca, serum levels of calcium; FGF23, fibroblast growth factor 23; NA, not applicable; P, serum levels of phosphate; PTH, parathyroid hormone; TmP/GFR, maximum rate of renal tubular reabsorption of phosphate per glomerular filtration rate; U_Ca_, urinary calcium excretion; U_P_ , urinary phosphate excretion; VDR, vitamin D receptor. Data from ref.^58^. ^a^Cave: prevalence of vitamin D deficiency was reported to be up to 50% in healthy children. ^b^Normal after restoration of P, but falsely reduced before restoration. ^c^PTH might be moderately elevated. ^d^Decreased relative to the serum phosphate concentration. ^e^Depending on the stage of chronic kidney disease.

### Genotype–phenotype correlation

Although XLH seems to be completely penetrant, its severity varies widely, even among family members, with no clear gender difference^55,65^. A large number of inactivating mutations in *PHEX* can cause XLH, and a genotype–phenotype correlation is not obvious^31^.

### Further patient work-up

Patients should be evaluated for the presence and severity of common and rare complications of XLH depending on their age (Table 1). In young children, dynamic tests or specific investigations, such as hearing evaluation or oral examination, are sometimes not feasible and can be delayed until the child reaches 3–5 years of age.

### Other causes of hypophosphataemic rickets

**Fig. 3:**
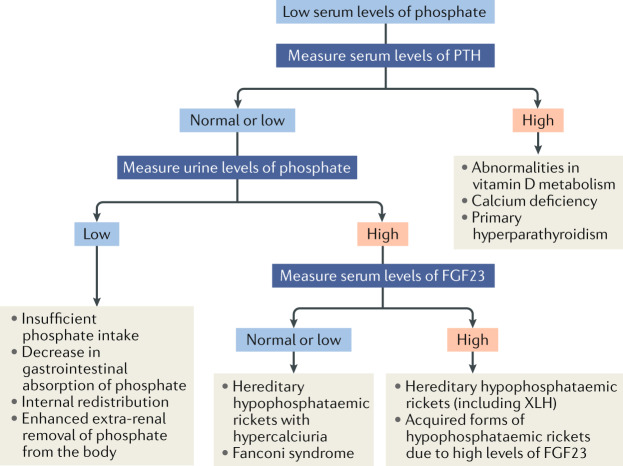
Algorithm for the evaluation of a child with rickets presenting with hypophosphataemia. The differential diagnoses are based on the mechanisms leading to hypophosphataemia — namely, high parathyroid hormone (PTH) activity, inadequate phosphate absorption from the gut or renal phosphate wasting. The latter may be due to either primary tubular defects or high levels of circulating fibroblast growth factor 23 (FGF23). Further details of individual entities can be found in Table 2. XLH, X-linked hypophosphataemia. Adapted with permission from ref.^57^, Springer Nature Limited (this material is excluded from the CC-BY-4.0 license).

XLH represents ~80% of all cases of hypophosphataemic rickets^39,43,45^. Other types of hypophosphataemic rickets have similar but not identical clinical and radiological features^58^. Other causes of hypophosphataemia must be considered in patients with a negative family history, male-to-male transmission or unusual clinical presentation, such as symptoms developing after the second year of life (autosomal dominant hypophosphataemic rickets or tumour-induced osteomalacia), acidosis, glucosuria, aminoaciduria or low molecular mass proteinuria (renal Fanconi syndrome), low urinary phosphate levels (dietary phosphate deficiency or impaired bioavailability) or hypercalciuria before starting treatment (hereditary hypophosphataemic rickets with hypercalciuria (HHRH)) (Table 2). The differential diagnoses are based on the mechanisms leading to hypophosphataemia — namely, high PTH activity, inadequate phosphate absorption from the gut or renal phosphate wasting. The latter might be the result of either primary tubular defects or high levels of circulating FGF23 (refs^66–68^) (Fig. 3; Table 2). Extended molecular genetic analysis can be helpful to establish the diagnosis in unclear cases of hypophosphataemic rickets^69–71^. Nutritional rickets and XLH might sometimes coexist, and diagnosis of XLH should be considered in someone otherwise thought to be vitamin D or calcium deficient if serum levels of phosphate do not improve after supplementation.

## Follow-up of patients with XLH

We suggest that patients should be seen at regular intervals by multidisciplinary teams organized by an expert in metabolic bone diseases (Box 2). The clinical, biochemical and radiological features and complications of XLH vary widely from patient to patient; therefore, treatment and monitoring should ultimately be tailored to the patient on the basis of their clinical manifestations, medical history, stage of development and the clinician’s professional judgement. The expert should liaise with the patient’s local health-care providers (general practitioners and/or paediatricians), radiologists, orthopaedic surgeons, physical therapists, rheumatologists and dentists. In addition, the following professions might be involved on the basis of individual patient needs: neurosurgeons, otolaryngologists (ENTs), ophthalmologists, orthodontists, dieticians, chiropodists, social workers and psychologists (Table 3).

**Table 3:** Summary of the recommendations for the follow-up of children and adults (both treated and untreated) with XLH

Examination	0–5 years	5 years to start of puberty (9–12 years)	Puberty^a^	Adults
Frequency of visits	Monthly to thrice monthly	3–6 months	3 months	6–12 months
Height, weight, IMD and ICD	✓	✓	✓	✓
Head circumference and skull shape	✓	NA	NA	NA
Presence of rickets, pain, stiffness and fatigue	✓	✓	✓	✓^b^
Neurological examination (consequences of craniosynostosis and spinal stenosis)	✓	✓	✓	✓
Musculoskeletal function, 6MWT^c^	Not feasible	Once a year	Once a year	Once a year
Orthopaedic examination	Once a year in the presence of significant leg bowing			Once a year^d^
Dental examination	Twice yearly after tooth eruption	Twice yearly	Twice yearly	Twice yearly
Hearing test	Not feasible	From 8 years: hearing evaluation if symptoms of hearing difficulties		
Serum levels of ALP (children), BAP (adults), calcium, phosphate, PTH and creatinine; eGFR	✓	✓	✓	✓
25(OH) vitamin D levels	Once a year	Once a year	Once a year	Once a year
Urine test: calcium:creatinine ratio^e^	Every 3 to 6 months on conventional treatment and burosumab treatment			
Fasting serum phosphate levels and TmP/GFR	• On burosumab treatment: every 2 weeks during the first month, every 4 weeks during the following 2 months and thereafter as appropriate • Titration period: between injections, ideally 7–11 days after last injection to detect hyperphosphataemia • After achievement of a steady state (which can be assumed after 3 months of a stable dose): preferentially directly before injections (children) or during the last week before the next injection (adults) to detect underdosing • Also measured 4 weeks after dose adjustment			
1,25(OH)_2_ vitamin D levels	Every 3 to 6 months in patients on burosumab treatment (analysed together with U_Ca_)			
Blood pressure	Twice yearly	Twice yearly	Twice yearly	Twice yearly
Renal ultrasonography	Every 1–2 years on conventional or burosumab treatment			
Left wrist and/or lower limbs radiographs	• If leg bowing does not improve upon treatment (children) • If surgery is indicated • Focused on any area of localized persistent bone pain • In case of short stature (bone age assessment)		In adolescents with persistent lower limb deformities when they are transitioning to adult care	NA
Dental orthopantogram	Not feasible	Based on clinical needs	Based on clinical needs	Based on clinical needs
Fundoscopy and brain MRI	If aberrant shape of skull, headaches or neurological symptoms	If recurrent headaches, declining school/cognitive performances or neurological symptoms		
Cardiac ultrasonography^f^	In presence of persistent elevated blood pressure (>95th percentile)			
QOL^g^	Not feasible	Every 2 years if available	Every 2 years if available	Every 2 years if available

6MWT, 6-minute walk test^77^; ALP, alkaline phosphatase; BAP, bone alkaline phosphatase; eGFR, estimated glomerular filtration rate^195,196^; ICD, intercondylar distance (reference values are given here^73^); IMD, intermalleolar distance; NA, not applicable; PTH, parathyroid hormone; QOL, quality of life; TmP/GFR, maximum rate of renal tubular reabsorption of phosphate per glomerular filtration rate. ^a^These examinations should also be performed at the time of transition to adult care. ^b^Also search for osteomalacia, pseudofractures, osteoarthritis and enthesopathy. ^c^If available. ^d^In symptomatic patients. ^e^Upper normal range (mol/mol): 2.2 (<1 years), 1.4 (1–3 years), 1.1 (3–5 years), 0.8 (5–7 years) and 0.7 (>7 years). ^f^According to international guidelines. ^g^Using age-appropriate and disease-appropriate QOL scales.

Box 2 Recommendations for follow-up of patients with XLH **Frequency and setting of visits**

- We suggest that patient care should be provided by multidisciplinary teams organized by an expert in metabolic bone diseases (grade D, weak recommendation)
- We suggest that children with X-linked hypophosphataemia (XLH) should be seen at least every 3 months during phases of rapid growth (infancy and puberty) or after initiation of therapy (grade C, weak recommendation)
- We suggest that patients demonstrating a positive response to treatment and/or in a stable condition should be seen at least every 6 months (grade C, weak recommendation)
- We suggest that adult patients should be seen every 6 months if receiving therapy or once a year if not treated with medications (grade C, weak recommendation)

**Follow-up of children with XLH (grade C, moderate recommendation)**

- We recommend measuring height, weight, head circumference (until the age of 5 years), intercondylar and intermalleolar distances and blood pressure
- We recommend calculating body mass index (BMI) and annual height velocity
- We recommend recording head shape and history of headaches, dental abscess or maxillofacial cellulitis, bone pain, fatigue and level of physical function
- We recommend that an orthopaedic assessment of the musculoskeletal system should be performed in the presence of lower limb deformity (varum or valgus or anteroposterior)
- We recommend searching for evidence of hearing loss, spine deformity and scoliosis, manifestations related to craniosynostosis, Chiari 1 malformation and/or intracranial hypertension and maxillary dysmorphosis
- We recommend assessing bone age to evaluate the growth potential in children >5 years old with growth impairment

**Follow-up of adults with XLH (grade C, moderate recommendation)**

- We recommend measuring height, weight and blood pressure and calculating BMI
- We recommend recording history of headaches, oral manifestations (including periodontal disease, dental abscess or maxillofacial cellulitis), musculoskeletal pain, pseudofractures, fatigue and level of physical function
- We recommend searching for evidence of hearing loss, enthesopathies, osteoarthritis, spine deformity and scoliosis, muscular deficiency, range of movement, manifestations related to Chiari 1 malformation and/or intracranial hypertension

**For all patients**

- We recommend twice-yearly dentist visits after tooth eruption to prevent and treat dental infections and periodontitis (grade C, moderate recommendation)
- We recommend monitoring of blood levels of alkaline phosphatase (ALP; total serum ALP levels in children and bone-specific ALP in adults), calcium, phosphate, creatinine, parathyroid hormone (PTH) and 25(OH) vitamin D (grade B, moderate recommendation). We recommend measuring calcium and creatinine levels in urine to calculate the urinary calcium:creatinine ratio in patients on conventional or burosumab treatment (grade B, moderate recommendation)
- In patients on burosumab treatment, we recommend monitoring fasting serum phosphate levels (grade B, moderate recommendation) together with tubular maximum reabsorption of phosphate per glomerular filtration rate (TmP/GFR) (grade B, weak recommendation) every 2 weeks during the first month of treatment, and every 4 weeks for the following 2 months and thereafter as appropriate; we also recommend measuring fasting serum phosphate level 4 weeks after dose adjustment (grade B, moderate recommendation) and suggest measuring 1,25(OH)_2_ vitamin D serum levels every 6 months analysed together with the urinary calcium excretion as safety parameters (grade B, weak recommendation)
- We recommend assessing disease severity through radiographs of the left wrist and/or knees in children who do not respond well to therapy or whose bone deformities worsen despite medical treatment, in children who may need orthopaedic surgery, in children who complain of unexplained bone pain or in adolescents with persistent lower limb deformities when they are transitioning to adult care. Radiographs should be standardized anterior–posterior standing long leg radiographs (utilizing low-dose radiation when feasible) to assess limb deformities, joint alignment and bone quality (grade B, moderate recommendation)
- In patients on conventional or burosumab treatment, we recommend kidney ultrasonography at least every 2 years in patients without nephrocalcinosis and at yearly intervals in patients with nephrocalcinosis and/or persistent hypercalciuria (grade C, moderate recommendation)
- We recommend cranial MRI (if possible including a black bone sequence to image the skull) in case of skull morphology that suggests craniosynostosis or clinical signs of intracranial hypertension (grade C, moderate recommendation)
- We suggest a dental orthopantomogram (radiograph of the upper and lower jaw and teeth) at 5 years of age and in adults with recent oral manifestations. Radiographs should be repeated on the basis of individual needs; retrocoronal and periapical radiographs and cone beam computed tomography can be used to detect and monitor endodontic, periodontal or peri-implant infections (grade D, weak recommendation)
- We do not recommend routine dual-energy X-ray absorptiometry (DXA) or peripheral quantitative computed tomography (pQCT) in patients with XLH for assessment of bone health (grade C, moderate recommendation)
- We suggest providing the contact details of patient association groups to patients, informing patients of scientific discoveries (including new therapies), supporting school and professional input and providing social support (grade D, weak recommendation)
- We suggest considering the 6-minute walk test (6MWT) and evaluating quality of life if facilities are available in patients from aged 5 years onwards at yearly or 2 yearly intervals (grade D, weak recommendation)

### Clinical evaluation

In children, signs or severity of rickets should be assessed at each visit, including measurement of intercondylar and/or intermalleolar distance, in addition to height and growth velocity^72,73^. With appropriate conventional therapy (that is, phosphate supplementation and treatment with active vitamin D), rickets should improve, leading to a reduction in limb deformity^8^, and height should increase by ~1 s.d. within 2–3 years when initiated in preschool-age children^8,52,74–76^. Often, lower limb deformity and joint alignment cannot be assessed fully with clinical measurements alone and radiographic assessment is helpful. Patients with substantial limb deformities should be evaluated by an orthopaedic surgeon with experience in metabolic bone disease. Such an evaluation should include an assessment of limb length and alignment (in both the coronal and sagittal planes) as well as the torsional profile of the lower limb.

Yearly assessment of the 6-minute walk test (6MWT) in patients >5–6 years might help to quantify the functional consequences of XLH on bone and muscles^77^. Patients should have at least twice-yearly dental examinations after tooth eruption, orthodontic evaluation around the age of 12 years and an extended dental evaluation with transition to adult care. The number of dental abscesses and episodes of acute oral infections (including maxillofacial cellulitis) should be recorded at each visit (as these are indirect indices of impaired tooth mineralization). Craniosynostosis should be considered in children up to the age of 5 years who have an insufficient increase in head circumference, abnormal head shape or neurological signs (including headache and vomiting as a result of increased intracranial pressure)^54,78,79^. The spine should be examined clinically for lordosis, kyphosis and/or scoliosis. In addition, bone and joint pain, disability and fatigue should be assessed by patient-reported outcomes.

### Biochemical follow-up

Serum level of ALP is a reliable biomarker of rickets activity and osteomalacia in children and adults^8,80–83^. Given that bone-specific ALP represents ~80–90% of total ALP in the serum of children, total ALP might be used in this population. In adults, bone-specific ALP is preferred given that ~50% of circulating ALP originates from hepatocytes^84^. When rachitic or osteomalacic bones are undertreated, ALP levels are elevated and urinary levels of calcium are usually low. By contrast, when rickets is healed, ALP levels tend to normalize and urinary calcium levels start to increase^7,8^. PTH should be measured regularly as secondary hyperparathyroidism is promoted by oral phosphate supplementation^64,85–88^. Suppressed PTH levels suggest that the dose of active vitamin D is disproportionately high compared with phosphate supplementation. Measurement of serum and urinary levels of calcium is required to evaluate the safety of active vitamin D. Spot urine samples are preferred as they are simple to perform, especially in young children. Alternatively, 24-hour urine collections can be performed in toilet-trained patients. We do not recommend regular measurement of serum levels of FGF23 in treated patients as it does not guide therapy^6,14,89,90^.

In patients treated with burosumab, fasting serum phosphate level is a biomarker of efficacy and should be monitored to titrate the treatment in children and to exclude hyperphosphataemia. In some patients, burosumab might initially normalize TmP/GFR while serum level of phosphate is still below the normal range owing to the high demand for phosphate of the bone. In this setting, an increase in burosumab dose will not necessarily result in improved bone healing. Therefore, we suggest that TmP/GFR should be analysed together with fasting serum phosphate levels as a measure of drug efficacy. Serum levels of 1,25(OH)_2_ vitamin D might increase under burosumab therapy; we suggest measuring these levels every 6 months and analysing them together with the urinary calcium excretion as safety parameters^20,91–93^.

### Radiographic and imaging follow-up

Radiographic evaluation is recommended in cases of persistent marked clinical or biochemical signs, for example, elevated levels of ALP despite adequate therapy. Enthesopathies, osteoarthritis and pseudofractures can be accurately diagnosed on plain radiographs and bone nuclear scans^17,26,94^. The EOS system (which is associated with 50–80% less irradiation than conventional radiography; EOS Imaging, Paris, France) enables 3D assessment of limb deformity^95^ and should be used whenever possible^95^. Renal ultrasonography is the preferred method to screen for nephrocalcinosis^86,96–99^.

A dental orthopantomogram (radiograph of the upper and lower jaw and teeth) is suggested at 5 years of age and in adults with recent oral manifestations and should be repeated on the basis of clinical needs.

In symptomatic adults and children (such as those with persistent headache, vomiting or abnormal skull shape), we recommend evaluation by brain and/or spinal MRI to exclude craniosynostosis, Chiari 1 malformation or syringomyelia. To avoid excessive irradiation from radiography or computed tomography (CT), the skull can be imaged through the black bone sequence on MRI, which provides a high image contrast between bone and other tissues^100^.

The clinical value of peripheral quantitative CT (pQCT) or bone mineral density (BMD) measurements by DXA (dual-energy X-ray absorptiometry) in patients with XLH is limited^101–103^. Both methods are unable to diagnose osteomalacia. Intriguingly, trabecular volumetric BMD was even found to be increased in paediatric patients with XLH^101–103^, which might reflect a compensatory mechanism owing to the soft bone.

Elevated arterial blood pressure, left ventricular hypertrophy and/or pathological electrocardiograms have been reported in some but not all studies evaluating the cardiovascular status of patients with XLH^104–110^. Therefore, we suggest at least yearly blood pressure measurements, with a more detailed work-up only in the presence of persistently elevated blood pressure.

## Conventional treatment in children

### Phosphate

Recommendations for conventional treatment in children with XLH are provided in Box 3. Oral phosphate supplements should always be provided together with active vitamin D, as phosphate alone promotes secondary hyperparathyroidism and thereby renal phosphate wasting^32,48,111^. Treatment doses vary according to age and severity of phenotype, and no consensus exists on the optimal dose of oral phosphate^7,8^. However, starting doses of 20–60 mg/kg body weight daily (0.7–2.0 mmol/kg daily) based on elemental phosphorus are recommended on the basis of the severity of the phenotype. Early treatment is associated with superior outcomes^18,26,32,49,50,75,112–115^. Healing of rickets, as evidenced by the normalization of ALP levels and radiological signs, is the initial aim in children^8,32,116^. Treatment also promotes growth, reduces bone pain, progressively corrects leg deformities and improves dental health^48,50,52,63,74,75,81–83,85,111,117^. In infants diagnosed before they develop bone changes, the goal of treatment is to prevent rickets. Serum phosphate levels increase rapidly after oral intake but return to baseline concentrations within 1.5 hours^63^. Thus, phosphate should be given as frequently as possible, for example, 4–6 times per day in young patients with high ALP levels, to maintain stable blood levels. Less frequent dosing (2–3 times daily) might improve adherence in adolescents. Fasting phosphate levels are not restored by oral phosphate supplements, and normalization of serum levels of phosphate is not a goal of conventional therapy^7,8^. Phosphate supplements are available as oral solutions, capsules or tablets containing sodium-based and/or potassium-based salts. Dosages should always be based on elemental phosphorus given that the phosphorus content largely differs between the available phosphate salts. Oral solutions containing glucose-based sweeteners should be used with caution given the dental fragility of these patients. Phosphate should not be given together with calcium supplements or foods with high calcium content, such as milk, as precipitation in the intestinal tract reduces absorption.

Box 3 Recommendations for treatment in children

- We recommend treating children with overt X-linked hypophosphataemia (XLH) phenotype with a combination of oral phosphorus (phosphate salts) and active vitamin D (calcitriol or alfacalcidol) as soon as diagnosis is established (grade B, moderate recommendation)
- We recommend an initial dose of 20–60 mg/kg body weight daily (0.7–2.0 mmol/kg daily) of elemental phosphorus in infants and preschool children, which should be adjusted according to the improvement of rickets, growth, alkaline phosphatase (ALP) and parathyroid hormone (PTH) levels (grade C, moderate recommendation)
- We recommend phosphate supplements should be taken as frequently as possible, for example, 4–6 times daily in young patients with high ALP levels. The frequency can be lowered to 3–4 times daily when ALP has normalized (grade B, moderate recommendation)
- We recommend a progressive increase in the dose of phosphate supplements in cases of insufficient clinical response but avoidance of doses >80 mg/kg daily (based on elemental phosphorus) to prevent gastrointestinal discomfort and hyperparathyroidism. If these adverse effects are present, treatment should be adjusted by decreasing the dose and/or increasing the frequency (grade C, moderate recommendation)
- We recommend the use of low doses in patients with mild phenotypes, for instance, infants diagnosed by family screening (grade C, moderate recommendation)
- We recommend an initial dose of calcitriol of 20–30 ng/kg body weight daily or alfacalcidol of 30–50 ng/kg body weight daily. Alternatively, treatment can be started empirically at 0.5 μg daily of calcitriol or 1 μg of alfacalcidol in patients >12 months old and adjusted on the basis of clinical and biochemical responses (grade C, moderate recommendation)
- To prevent nephrocalcinosis, we recommend keeping calciuria levels within the normal range and avoiding large doses of phosphate supplements; we suggest measures that decrease urinary calcium concentration, excretion and/or crystallization if necessary, including regular water intake, administration of potassium citrate and limited sodium intake (grade C, moderate recommendation)
- With respect to secondary hyperparathyroidism, we recommend the following:
  - Patients on conventional treatment with elevated PTH levels should be managed by increasing the dose of active vitamin D and/or decreasing the dose of oral phosphate supplements (grade C, moderate recommendation)
  - Treatment with calcimimetics might be considered in patients with persistent secondary hyperparathyroidism despite the above-mentioned measures (grade D, weak recommendation). Cinacalcet should be used with caution in XLH, as it has been associated with severe adverse effects, namely, hypocalcaemia and increased QT interval (grade X, strong recommendation)
  - Parathyroidectomy should be considered in case of tertiary hyperparathyroidism (persistent hypercalcaemic hyperparathyroidism) despite optimized active vitamin D and cinacalcet therapy (grade C, moderate recommendation)

- We suggest supplementing patients with native vitamin D (cholecalciferol or ergocalciferol) in case of vitamin D deficiency (grade C, weak recommendation)
- We do not recommend routine calcium supplementation in children with XLH, but a dietary evaluation of daily calcium intake should be performed (grade D, weak recommendation)
- We recommend that treatment plans should be discussed in a multidisciplinary team setting before surgery; we also suggest that active vitamin D supplementation should be decreased or stopped if patients are immobilized for a long period; therapy should be restarted as soon as the patient resumes walking (grade D, weak recommendation)

### Active and native vitamin D

Active vitamin D (calcitriol or alfacalcidol) is given in addition to oral phosphate supplements in order to counter calcitriol deficiency, prevent secondary hyperparathyroidism and increase phosphate absorption from the gut. The optimal dose varies from patient to patient. Requirements are generally higher during early childhood and puberty (growth phases)^51^, and the dose can be adjusted on the basis of serum levels of ALP and PTH and urinary calcium excretion. Large doses of active vitamin D promote growth and bone healing but are associated with an increased risk of hypercalciuria and nephrocalcinosis^96–99,118^. Conversely, insufficient doses of active vitamin D are usually associated with low intestinal calcium absorption, low urinary calcium excretion, persistent rickets and high ALP and/or PTH levels. Calcitriol can be given in one or two doses per day, whereas alfacalcidol should be give once per day owing to its longer half-life^8^. The equivalent dosage of alfacalcidol is 1.5–2.0 times that of calcitriol on the basis of studies in patients with secondary hyperparathyroidism due to chronic kidney disease and observations in patients with XLH^8,119^. This difference is probably because of the roughly twice higher oral bioavailability of calcitriol than alfacalcidol. A single evening dose might help prevent excessive calcium absorption after food intake and thus hypercalciuria^120^. Several other vitamin D analogues are also available^62,117,121,122^. As in healthy children, 25(OH) vitamin D deficiency in children with XLH should be corrected by supplementation with native vitamin D.

### Calcium supplements

Nutritional calcium intake should be kept within the normal range for age. Supplements are not recommended given that bone mass and mineral content are usually not decreased and because of the potential risk of hypercalciuria^101,103,123,124^.

### Adverse effects of conventional treatment

Conventional treatment with phosphate supplementation and active vitamin D might increase calciuria and thereby promote nephrocalcinosis, which has been reported in 30–70% of patients with XLH^3,48,56,87,96–99,118^. Several reports suggest positive associations between daily oral phosphate doses and the risk of developing nephrocalcinosis, whereas the relationship with active vitamin D therapy and/or with the presence of hypercalciuria has been observed less frequently^3,48,96,97,99,118,125^. Hydrochlorothiazide decreases calciuria in XLH^86^, and potassium citrate might help prevent calcium precipitation, especially in patients with low urinary citrate levels; however, alkalinization of urine increases the risk of phosphate precipitation. Therefore, potassium citrate should be used with caution in XLH.

Secondary hyperparathyroidism, which might aggravate phosphaturia and promote bone resorption, results from the long-term stimulation of parathyroid cells by FGF23 and phosphate supplements and from the suppression of 1,25(OH)_2_ vitamin D levels by FGF23, especially in patients not treated with active vitamin D^62,64,86–88,104,126–128^. Conversely, suppressed PTH levels secondary to excessive vitamin D therapy and/or insufficient oral phosphate intake might decrease bone turnover and compromise rickets healing and growth. Thus, therapies should be adjusted to keep PTH levels within the normal range (10–65 pg/ml in children and adults). In patients with XLH, adjuvant therapy with a calcimimetic (for example, cinacalcet) decreases serum levels of PTH and FGF23 and increases TmP/GFR^126,129,130^. Therefore, if PTH levels do not normalize after optimizing active vitamin D (dose increase) and phosphate treatment (dose reduction), cinacalcet might be considered together with close monitoring^130^. However, cinacalcet is not licensed for this indication and has been associated with severe adverse effects — namely, hypocalcaemia and increased QT interval^131^. To date, no evidence suggests that burosumab can revert persistent hyperparathyroidism. Therefore, parathyroidectomy should be considered in patients with tertiary hypercalcaemic hyperparathyroidism.

## Burosumab in children with XLH

In February 2018, the European Medicines Agency (EMA) granted a conditional marketing authorization in the European Union for burosumab for the treatment of XLH with radiographic evidence of bone disease in children ≥1 year of age and in adolescents with a growing skeleton^23^. In April 2018, the US Food and Drug Administration (FDA) granted approval of burosumab to treat adults and children ≥1 year with XLH^24^. These decisions were based on the results of trials testing burosumab in children with severe XLH and in adults with skeletal pain associated with XLH and/or osteomalacia. Serum levels of phosphate, TmP/GFR, the severity of rachitic lesions in children (based on radiography images) and osteomalacia in adults (based on radiography images and bone histomorphometry) were chosen as primary end points. In children, the dose of burosumab was initially titrated against serum levels of phosphate, targeting empirical levels ranging from 1.1 to 1.6 mmol/l, whereas adult patients received a fixed weight-related dose^19,22^.

Currently only the data submitted to the regulatory agencies and published in peer review journals are available^19,132,133^. Burosumab is an expensive drug, and data on cost-effectiveness and long-term outcome are pending^134,135^. Thus, conclusive recommendations on the use of burosumab are premature. However, given the severity of the disease in some patients and the encouraging results that have prompted the EMA and FDA to grant authorization, preliminary recommendations are provided (Box 4).

Two open-label uncontrolled trials testing burosumab in a total of 65 children aged 1–12 years with severe XLH demonstrated that in the short term (12–16 months), burosumab resulted in the following outcomes^19,132,133^: a statistically significant increase in TmP/GFR and consequently raised serum phosphate levels into the lower end of the age-related normal range, with increased 1,25(OH)_2_ vitamin D levels; a significant reduction in the severity of rickets (as measured by the Rickets Severity Score (RSS) and the Radiographic Global Impression of Change (RGI-C)); a significant improvement in physical ability (as measured by walking distance in the 6MWT); and a significant reduction in patient-reported pain and functional disability (as measured by the use of the Pediatric Orthopedic Society of North America Outcomes Data Collection Instrument).

The most common adverse reactions observed with burosumab were injection-site reactions, headache and pain in the extremities. Two weekly doses were superior to four weekly doses with respect to normalization of serum levels of phosphate and radiological improvement of rickets. Conventional treatment should be stopped at least 1 week before the start of burosumab therapy for wash-out and to prove that fasting serum phosphate levels are below the normal reference for age.

The EMA and FDA authorizations approved a starting dose of 0.4 mg/kg body weight and 0.8 mg/kg body weight, respectively, given every 2 weeks^132,133^. We suggest starting with a dose of 0.4 mg/kg body weight as this dose might be sufficient. The dose should be titrated in increments of 0.4 mg/kg body weight in order to raise fasting serum phosphate levels within the lower end of the normal reference range for age, with a maximum dosage of 2.0 mg/kg body weight (maximum dose 90 mg). In paediatric trials, the average maintenance dose was 1 mg/kg body weight^19^.

Pharmacokinetic and pharmacodynamic studies showed similar results in children and adults, with a drug half-life of ~19 days and peak serum concentrations of burosumab at 7–11 days after injection, which paralleled with the increase in serum levels of phosphate and TmP/GFR, thus supporting a direct pharmacokinetic–pharmacodynamic relationship^133^. We therefore suggest monitoring fasting serum phosphate levels during the titration period between injections, ideally 7–11 days after the last injection, to avoid inadvertently causing hyperphosphataemia. After achievement of a steady state, which can be assumed after 3 months of a stable dosage, we suggest monitoring serum levels of phosphate preferentially directly before injections to detect hypophosphataemia. Burosumab should not be adjusted more frequently than every 4 weeks, and longer intervals of at least 2 months are suggested. Burosumab must not be given when phosphate levels are within the age-related normal reference range before initiation of treatment or in the presence of severe renal impairment (as these patients are at risk of developing hyperphosphataemia). Targeting fasting serum phosphate in the lower end of the normal reference range for age is probably the safest approach to limit the risk of ectopic mineralization.

Box 4 Recommendations for burosumab in children with XLH

- If available, we recommend considering burosumab treatment in children with X-linked hypophosphataemia (XLH) ≥1 year and in adolescents with growing skeletons in the following situations: radiographic evidence of overt bone disease and disease that is refractory to conventional therapy; or complications related to conventional therapy; or patient’s inability to adhere to conventional therapy, presuming that adequate monitoring is feasible (grade B, moderate recommendation)
- In children, we recommend a starting dose of burosumab of 0.4 mg/kg body weight, given subcutaneously every 2 weeks (grade B, moderate recommendation)
- We recommend titrating burosumab in 0.4 mg/kg increments to raise fasting serum phosphate levels within the lower end of the normal reference range for age to a maximum dosage of 2.0 mg/kg body weight (maximum dose 90 mg) (grade B, moderate recommendation)
- Burosumab should not be adjusted more frequently than every 4 weeks (grade B, moderate recommendation)
- We suggest monitoring of fasting serum phosphate levels during the titration period between injections, ideally 7–11 days after last injection, to detect hyperphosphataemia; after achievement of a steady state, which can be assumed after 3 months of a stable dosage, fasting serum phosphate levels should be assessed preferentially directly before injections to detect underdosing (grade B, weak recommendation)
- The dose should be discontinued if fasting serum phosphate level is above the upper range of normal. Burosumab can be restarted at approximately half of the previous dose when serum phosphate concentration is below the normal range (grade B, moderate recommendation)
- We recommend that burosumab must not be given in conjunction with conventional treatment, when fasting phosphate levels are within the age-related normal reference range before initiation of treatment or in the presence of severe renal impairment (grade X, moderate recommendation)

## Growth hormone

Final heights are reduced in up to 60% of patients with XLH despite conventional treatment^8,48–50,52,56,75,76,136–139^. Administration of recombinant human growth hormone (rhGH) resulted in a sustained increase in age-standardized height during treatment periods of up to 3 years, and prepubertal children responded better to rhGH than pubertal patients^140^. Administration of rhGH was associated with a transient increase in serum levels of phosphate and PTH. A 3-year randomized controlled trial in severely short children with XLH showed substantial growth response in comparison to controls individuals, without aggravation of body disproportion^141^. However, long-term follow-up of the same study failed to show significant benefits on the adult height, whereas in another study mean final height was significantly higher than in patients who did not receive rhGH^142,143^ (Box 5).

Box 5 Recommendations for growth hormone

- We do not recommend routine treatment with recombinant human growth hormone (rhGH) for patients with X-linked hypophosphataemia (XLH) (grade C, weak recommendation)
- Children with short stature might be considered for rhGH therapy, provided that levels of alkaline phosphatase and parathyroid hormone are well controlled (grade C, weak recommendation)

## Conventional treatment in adults

Treatment is recommended in symptomatic adult patients with XLH — that is, those with musculoskeletal pain, pseudofractures, dental issues, planned orthopaedic or dental surgery or biochemical evidence of osteomalacia with an increase in serum levels of bone-specific ALP (Box 6). Conventional treatment with active vitamin D and phosphate improves pain, osteomalacia and oral health (with respect to periodontitis and the frequency of dental abscesses) but does not prevent or improve hearing loss or enthesopathies.

In addition to oral health^18^, little evidence suggests that starting or continuing treatment in asymptomatic adults improves outcomes^8,16,17,56,122,144–146^. Taking daily active vitamin D and at least twice-daily oral phosphate supplements is burdensome for many adults and has potential adverse effects^16–18,26,56,113,114,122,144^.

Calcitriol or alfacalcidol doses that are usually prescribed in adults range from 0.50 to 0.75 μg daily for calcitriol and 0.75–1.5 μg daily for alfacalcidol. Phosphate supplements, available as oral solutions, capsules or tablets, containing sodium-based or potassium-based salts, should be given at 750–1,600 mg daily (based on elemental phosphorus) in 2–4 divided doses^26,82,83,114,115^. To avoid gastrointestinal adverse effects, the dose of phosphorus should be increased gradually. Theoretically, potassium-based phosphate salts could decrease the risk of hypercalciuria compared with sodium-based preparations. Taking active vitamin D, which increases calcium absorption, in the evening might reduce intestinal calcium absorption^3,16,17,26,82,83,86,87,96,99,114,122,124,144,147^. Vitamin D deficiency should be corrected as in the general population. Thiazide diuretics have been suggested to increase renal calcium reabsorption and to enhance bone mineralization; however, the long-term effects of this treatment are not known, as discussed in the treatment for children section^98,148^. Normal calcium intake (minimum 1g per day) and a low-sodium diet are recommended to reduce calciuria and support weight control.

Pregnancy is a critical moment for bone health. Therefore, during pregnancy, 25(OH) vitamin D levels should be monitored and adjusted. Phosphate supplementation might require higher dosages, up to 2,000 mg daily. Most patients already on therapy will simply continue their treatment. We also suggest considering conventional therapy for women with XLH who are not on therapy at the time of conception. All treated pregnant women should undergo close biochemical monitoring. Women are encouraged to breastfeed if they want to, regardless of treatment. Conventional therapy is suggested in lactating women to prevent bone loss^149^.

Orthopaedic procedures are usually indicated to correct deformity (both angular and torsional) and for the treatment of pathological fractures. In adults, these conditions are unlikely to improve with medical management alone, which should nonetheless always accompany surgical management^16,144,150–152^. In patients undergoing orthopaedic surgery, therapy might need to be discontinued if long-term bed rest and/or non-weight-bearing mobilization is anticipated to avoid hypercalciuria and/or hypercalcaemia due to increased bone resorption^80,99^.

Box 6 Recommendations for conventional treatment in adults

- We recommend treatment in symptomatic adults with X-linked hypophosphataemia (XLH) by active vitamin D together with oral phosphorus (phosphate salts) to reduce osteomalacia and its consequences and to improve oral health (grade B, moderate recommendation)
- We suggest treating pregnant and lactating women with active vitamin D in combination with phosphate supplements if needed (grade D, weak recommendation)
- We do not recommend routine treatment of asymptomatic adults with XLH (grade C, moderate recommendation)
- We recommend using substantially lower doses of active vitamin D and oral phosphate than are used in children (grade C, moderate recommendation). We recommend a dose range of 750–1,600 mg daily (based on elemental phosphorus) for phosphate and of 0.50–0.75 and 0.75–1.5 μg daily for calcitriol and alfacalcidol, respectively (grade C, weak recommendation)
- We recommend reducing doses of active vitamin D in patients in whom long-term immobilization is anticipated, to prevent hypercalciuria and hypercalcaemia (grade D, weak recommendation)
- We recommend stopping phosphate supplements in patients with markedly increased parathyroid hormone levels (grade C, moderate recommendation)
- We suggest that active vitamin D might be given without phosphate supplements to adult patients with secondary hyperparathyroidism if careful follow-up is provided (grade D, weak recommendation)
- We suggest supplementing patients with native vitamin D (cholecalciferol or ergocalciferol) in case of vitamin D deficiency; we also suggest ensuring normal calcium intake (grade C, weak recommendation)

## Burosumab in adult patients

One open-label, uncontrolled trial and one randomized, double blind, placebo-controlled study (including a total of 148 patients) have investigated burosumab in adults with skeletal pain associated with XLH and/or osteomalacia^25,132,133^. Short-term treatment (6–12 months) with burosumab was associated with the following outcomes^25,132,133^: significantly increased TmP/GFR and consequently raised serum levels of phosphate into the lower normal range and increased 1,25(OH)_2_ vitamin D levels; healed osteomalacia and accelerated healing of active fractures and pseudofractures; and significantly reduced stiffness (as measured by the Western Ontario and the McMaster Universities Osteoarthritis Index (WOMAC) stiffness subscale). By contrast, reductions in WOMAC physical function subscale and the Brief Pain Inventory score did not achieve statistical significance when compared with placebo.

Notably, all studies to date in adults with XLH took place in moderately to severely affected patients, making extrapolation to real-world patients difficult as many real-world patients might have mild disease. The reported adverse events were similar to those observed in the paediatric trials (that is, injection-site reactions, headache and pain in the extremities). In the USA, the FDA approved a dose of burosumab of 1 mg/kg body weight, with a maximum dose of 90 mg, given subcutaneously every 4 weeks^132^. General monitoring during burosumab treatment is given in Table 3. Owing to the pharmacokinetic and pharmacodynamic characteristics of burosumab, we suggest initially monitoring fasting serum phosphate levels between injections, ideally 7–11 days after the last injection, to avoid inadvertently causing hyperphosphataemia (Box 7). After achievement of a steady state, which can be assumed after 3 months, we suggest measuring serum levels of phosphate, preferentially during the last week before the next injection, to detect underdosing^133^. Otherwise, the contraindications and modifications in patient monitoring are the same as in children.

Box 7 Recommendations for burosumab treatment in adults

- If available, we recommend considering burosumab treatment in adults with X-linked hypophosphataemia (XLH) with the following features: persistent bone and/or joint pain due to XLH and/or osteomalacia that limits daily activities; pseudofractures or osteomalacia-related fractures; and insufficient response or refractory to conventional therapy (grade B, moderate recommendation)
- We also recommend considering burosumab treatment if patients experience complications related to conventional therapy (grade D, weak recommendation)
- We recommend a starting dose of burosumab of 1.0 mg/kg body weight (maximum dose of 90 mg) given subcutaneously every 4 weeks (grade B, moderate recommendation)
- We suggest initial monitoring of fasting serum phosphate levels between injections, ideally 7–11 days after the last injection to detect hyperphosphataemia; after achievement of a steady state, which can be assumed after 3 months of a stable dosage, serum levels of phosphate should be assessed during the last week before the next injection to detect underdosing (grade B, weak recommendation)
- The dose should be discontinued if fasting serum phosphate level is above the upper limit of normal. Burosumab can be restarted at approximately half of the previous dose when serum phosphate concentration is below the normal range (grade C, moderate recommendation)
- Burosumab must not be given together with conventional treatment, in patients with phosphate levels within the age-related normal reference range before initiation of treatment or in the presence of severe renal impairment (grade X, moderate recommendation)

## Musculoskeletal symptoms

Muscle strength is considerably lower in patients with XLH than in healthy control individuals, without changes in muscle cross-sectional area^102,153,154^. Enthesopathies are prevalent in adult XLH and are usually detectable by the third decade of life; conventional therapy does not seem to prevent or treat these complications^26,155,156^. Musculoskeletal symptoms represent a high burden in adults with XLH, including stiffness due to joint involvement and/or the presence of enthesopathies, muscle weakness, fatigue and physical deconditioning and pain. These symptoms result in mobility impairment, reduced physical activity and reduced quality of life, which is a strong rationale for prescribing non-pharmacological treatments and self-management^17^. The goals of physical therapy are to provide pain relief, to improve physical function and fitness and to reduce XLH-related disability (Box 8). To date, no disease-specific recommendations exist for physical therapy in patients with XLH, and programmes are based on recommendations of physical therapies for individuals with knee or hip osteoarthritis.

Box 8 Recommendations for musculoskeletal treatment

- We recommend interventions aimed at reducing bone and joint pain, deformity, stiffness, muscular weakness and improving walking distance and physical function. These interventions include nonspecific measures including the use of analgesics (for example, short periods of use of nonsteroidal anti-inflammatory drug (NSAIDs)), intra-articular joint infiltrations (in the presence of degenerative changes), physiotherapy, rehabilitation, physical activity and non-pharmacological treatment of pain (grade D, weak recommendation).

## Orthopaedic management of XLH in children

Osteotomies performed early in childhood to treat deformity have historically been associated with a high recurrence rate and complications^11,157–159^. Currently, with improvements in medical care, limb deformity improves in most patients, persists in others and progresses to a severe deformity in a minority^9,10,52,160^. Surgical approaches now include the less invasive techniques of guided growth surgery performed early in childhood, which contrast with the active delay of corrective surgery using complex osteotomies until the patient is skeletally mature^9,160^. In guided growth surgery, a small metal plate is placed on the medial or lateral surface of the bone (for valgus or varus deformities) at the level of the physis (growth plate). A screw is placed into the bone at either side of the growth plate, and the device then acts as a tether to growth near the plate but not at the opposite side of the bone. Thus, with time, the differential growth allows the alignment to improve, and when the bone is straight, the plate is removed and normal growth continues.

This change in both medical and surgical treatment philosophies makes it difficult to make firm recommendations at this stage^161–164^ (Box 9).

Box 9 Recommendations for orthopaedic management in children with XLH

- We do not recommend the use of casts or insoles for the management of lower limb deformity in children with X-linked hypophosphataemia (XLH) (grade C, moderate recommendation)
- We suggest emphasizing the importance of weight-bearing exercise, maintenance of joint range and maximizing strength and endurance (grade D, weak recommendation)
- We do recommend physiotherapy following surgery or in case of decreased range of movement, muscle weakness, fatigue, instability or physical deconditioning (grade D, weak recommendation)
- We recommend that elective surgical treatment should be performed only in children in whom medical treatment has been maximized for at least 12 months (grade C, moderate recommendation)
- We suggest that surgery should be performed by a surgeon with expertise in metabolic bone diseases (grade B, moderate recommendation)
- We suggest that persisting deformity (mechanical axis deviation Zone 2 or greater) despite optimized medical treatment and/or the presence of symptoms interfering with function should be considered for surgical treatment (grade C, weak recommendation)
- We recommend that the age of the child should be considered as an important factor in the decision-making process: guided growth techniques depend on the remaining growth potential of the child and must therefore be carried out at least 2–3 years before skeletal maturity (age 14 in girls and age 16 in boys), whereas the complications associated with osteotomy reduce when the surgery is performed later in childhood or after skeletal maturity (grade C, moderate recommendation)
- We recommend that emergency surgical treatment such as fracture fixation should occur when necessary (grade B, moderate recommendation)
- We suggest that, following surgery, regular clinical and functional assessments should be made, including radiography, at 12 months post-surgery, or earlier if the bone deformity worsens and/or there is clinical concern. Further assessments should follow intermittently until skeletal maturity (grade C, moderate recommendation)

### Casts, insoles and physiotherapy

Evidence does not support the use of insoles or casts to improve lower limb deformity associated with XLH. Insoles cannot improve the position of a flat foot if this is distal to a valgus knee. Physiotherapy (in terms of a general strengthening and/or a gait education programme) might be helpful, especially after surgery.

### Orthopaedic surgery

The radiographic indications for elective surgical treatment include, in the coronal plane, a deviation of the mechanical axis into zones 3 or 4 (refs^160,165^). In children who are still growing, a mechanical axis that is progressive through zone 2 despite optimized medical care might also merit treatment^160^. Treatment at this stage is often simpler, more reliable and less prone to complications than treatment when patients have stopped growing. Substantial deformity might also exist in the sagittal and torsional planes. Deformity in three planes increases the difficulty of any surgical intervention, but often the coronal deformity dominates and correction of this plane improves the overall picture^160,165,166^.

The aim of surgical treatment is that at skeletal maturity the lower limbs are of equal length, well aligned (that is, with neutral lower limb mechanical axes and horizontal knee and ankle joints) and with mobile, comfortable joints. If possible, this goal should be achieved with the minimum amount of surgery, no complications, minimal time off school and/or activities and no functional loss.

Lower limb deformities can be treated by performing osteotomies at the site or sites of major deformity and correcting in all three planes. The osteotomy could result in acute correction of the deformity with internal fixation or gradual correction of deformity using external fixation techniques^9,117,122,159,160,167^. Such surgery is associated with notable rates of recurrence and of complications, especially in young children and in patients with poor metabolic control. In one study, 57% patients (28 of 49) experienced at least one complication of surgery, with recurrent deformity in 29% of patients^9^. Therefore, delaying surgical treatment for residual deformity until skeletal maturity might be prudent. Rarely, major deformities can induce severe knee instability, and these patients should undergo osteotomies before they stop growing.

Guided growth techniques have been gaining popularity^165^. This surgical strategy should commence early after 12 months if deformity persists despite maximized medical therapy. The rationale for this therapy is to correct the deformity at the physis before significant diaphyseal deformity develops. With correction of the mechanical axis, the forces directed across the physes and the joints are normalized, thus promoting normal growth. This technique does not require immobilization. The limitation of this strategy is that it provides only uniplanar coronal correction without correction of the tibial medial torsion. Torsional alignment often improves simultaneously. Both varus and valgus deformities correct readily and rapidly, but genu varum might respond less in adolescents^160^. In growing children, guided growth techniques might lead to overcorrection if the plates are left in situ for too long. Rebound deformity after plate removal has been reported, although rarely^12,160,168,169^.

Whether or not deformity correction at the knee results in some correction distally at the ankle is debated^160,168^. If early treatment could reduce deformity at distal joints, then the overall requirement for surgical procedures might be reduced. Similarly, if the limb alignment overall is satisfactory and the joints are horizontal, it is unclear as to whether residual minor diaphyseal deformity or torsional deformity causes significant functional difficulties.

### Assessment of surgical outcomes

Postoperative assessment of the axis correction should be documented at 12 months. Functional assessment should be performed according to the World Health Organization (WHO) International Classification of Function using tools such as the Pediatric Outcomes Data Collection Instrument (PODCI) or the 6MWT^77,170^. As patients transition to adult services, a full orthopaedic clinical and radiographic assessment enables definition of any residual deformity and facilitates appropriate follow-up arrangements.

## Dental health

### Endodontic infections

Children and adult patients with XLH might present with spontaneous endodontic infections on apparently intact teeth^18,114,115,171^. Dental abscesses can develop on deciduous as well as on permanent teeth^16,171^. The endodontic infection might be asymptomatic for months or years or it might evolve into dental abscesses, causing pain and swelling. Spread to surrounding anatomical structures causes maxillofacial cellulitis.

Dental complications in patients with XLH are secondary to poorly mineralized dentin. On dental radiographs, the pulp chambers of deciduous or permanent teeth are larger than usual with long pulp horns extending to the dentino–enamel junction^114,172^. XLH is not associated with increased susceptibility to caries^114,115,171^.

### Periodontitis

The frequency and severity of periodontitis is increased in adult patients with XLH and can lead to tooth loss. Cementum thickness, a central component of the periodontium, is reduced and associated with mineralization defects of the alveolar bone, which impairs the attachment of the ligament fibres^18,171^. On radiographs from adult patients, the lamina dura might be absent and the alveolar bone around teeth is frequently reduced^18,173^.

### Prophylaxis

Conventional treatment improves dentin mineralization, reduces the number of dental abscesses and decreases the frequency and severity of periodontitis^18,114^ (Box 10). Reducing the size of the pulp chambers, as demonstrated by dental radiographs, is a useful sign of successful treatment. Continued supplementation through adulthood may be beneficial, as the number of dental abscesses and severity of periodontitis vary according to the percentage of adult life with treatment^18,26^.

To prevent bacterial invasion of the dentin and pulp via enamel microcracks, we suggest sealing the occlusal surfaces of both deciduous and permanent teeth in children with XLH^174^. This approach can be achieved with flowable resin composite placed in the pits and fissures of the occlusal surface and should be performed regularly as soon as the eruption of the tooth allows acceptable isolation of the occlusal surface.

Acute abscesses might require antibiotic treatment depending on the extent and severity of the infection. For deciduous teeth, the decision to extract or treat endodontically will depend on the extent of the infection, recurrence and the expected timing of normal exfoliation of the permanent tooth. For permanent teeth, endodontic treatment or re-treatment of the tooth are the preferred options, although healing after endodontic treatment might not be as favourable as in healthy patients.

Prevention, treatment and supportive care of periodontitis in adult patients should follow standard management, as in the general population. Treatment of periodontitis should aim to decrease gingival inflammation and suppress periodontal pockets. The opportunity to resume or start conventional medical treatment should be discussed in the presence of periodontitis^18^.

Box 10 Recommendations for management of dental health

- In children and adults with ongoing oral manifestations, we recommend treatment with active vitamin D and phosphate supplementation to improve dentin mineralization, reduce the number of dental abscesses and reduce the severity of periodontitis (grade B, moderate recommendation)
- In children, in addition to standard preventive care, we recommend dental visits every 6 months and suggest sealing pits and fissures with flowable resin composite on both temporary and permanent teeth as soon and as frequently as required (grade C, weak recommendation)
- We suggest a thorough clinical investigation searching for pulp necrosis (colour changes, fistula, swelling, abscess, cellulitis or pain) and performing retrocoronal and/or periapical radiographs or orthopantomogram to search for enlarged pulp chambers and periapical bone loss depending on findings from a clinical examination (grade B, weak recommendation)
- We suggest optimizing conventional medical treatment of X-linked hypophosphataemia before initiation of orthodontic treatment (grade C, moderate recommendation)
- In adults, we recommend twice-yearly visits to perform conventional supportive periodontal therapy, which should include periodontal risk assessment and supragingival and subgingival debridement if necessary (grade B, moderate recommendation)
- In adults, we suggest that dental implant surgery should be performed after 3–6 months of medical treatment, which should be continued for 6 months following implant surgery; healing time should be extended up to 6 months (grade D, weak recommendation)

### Orthodontic and implant treatments

Children with XLH often present with delayed dental development, abnormal eruption patterns, increased frequency of specific malocclusions (including maxillary retrognathism) and impacted or dystopic maxillary canines^175–177^. Orthodontic treatment triggers movement of the teeth and extensive remodelling of the alveolar bone. Without conventional treatment, the outcomes of orthodontic treatment are unpredictable. The high rate of tooth loss secondary to endodontic infections and periodontitis often leads to the need for dental implants in adults with XLH^178,179^. Standard dental surgical protocols in adults with XLH who are not receiving conventional treatment result in decreased success rates compared with healthy control individuals^180^.

## Hearing loss

Impaired hearing has been observed in patients with XLH as early as 11 years of age, including subjective hearing loss, episodic tinnitus, deafness and vertigo^181,182^. Symptomatic patients present with generalized osteosclerosis and thickening of the petrous bone, and moderate internal auditory meatus narrowing, particularly in its mid-portion, compared with healthy control individuals^181,182^. Treatment is similar as for other causes of hearing loss and includes hearing aids, prevention of noise exposure and avoidance of ototoxic drugs (Box 11).

Box 11 Recommendations for management of hearing

- We suggest informing patients and families that hearing problems might occur and that any suspicion of hearing impairment should be investigated thoroughly (grade D, weak recommendation)
- We suggest treating hearing impairment similarly to other causes of peripheral hearing loss, with hearing aids, prevention of noise exposure and avoidance of ototoxic drugs (grade D, weak recommendation)

## Neurosurgical complications

### Craniosynostosis

Recommendations for the management of neurosurgical complications can be found in Box 12. Craniosynostosis can occur as early as 1 year of age and usually involves an abnormal fusion of the sagittal suture leading to a dolichocephalic conformation of the head with a reduced cranial index^54,79^. When systematically investigated with skull radiographic examination, suture fusion is identified in ~60% of children with XLH^79,183^. Craniosynostosis should be suspected in the presence of signs of intracranial hypertension, such as headache, neck pain or papilledema^54,78,79,184,185^. However, children might be asymptomatic, despite increased intracranial pressure^78,79,185^. An abnormal shape of the skull without any clinical symptoms does not necessarily mandate performing an MRI, and decisions should be made on an individual basis.

Box 12 Recommendations for management of neurosurgical complications

- We suggest a yearly basic neurological assessment, but we do not recommend further investigations in asymptomatic patients with X-linked hypophosphataemia (XLH) (grade C, weak recommendation)
- We suggest that patients and families should be informed that neurosurgical complications might occur and that any concerns about central nervous system function should be reported and addressed promptly (grade C, weak recommendation)
- We recommend a complete evaluation with fundoscopy and brain or skull imaging in any patient with XLH presenting with a skull morphology suggestive of craniosynostosis or clinical symptoms of intracranial hypertension, lower brainstem compression or compression of the upper cervical cord (suggesting a Chiari 1 malformation) (grade C, moderate recommendation)

## Chiari type 1 malformation

Chiari type 1 malformation, which causes prolapse of the cerebellar tonsils through the foramen magnum, is detected in 25–50% of children with XLH by use of cranial MRI or CT^54,79,183,186^. Most cases are asymptomatic, but compression of the lower brainstem and upper cervical cord might cause symptoms and/or result in syringomyelia requiring surgical correction. Symptoms can include occipital or neck pain exacerbated by Valsalva manoeuvres, peripheral motor and/or sensory defects, clumsiness, hyporeflexia or hyperreflexia, respiratory irregularities and central apnoeas and lower cranial nerve dysfunction^54,79^.

## Lifestyle

Evidence suggests that glucose and lipid metabolism might be compromised in individuals with XLH, resulting in obesity^127,128,187,188^. Adolescent and adult patients with XLH are prone to develop obesity partly because rheumatological and chronic bone complications decrease the propensity of patients to exercise^16,17,56,109,113,189^ (Box 13).

Box 13 Recommendations for lifestyle

- We recommend that physical activity in patients with X-linked hypophosphataemia should be supported and adapted to the patient’s ability. All sports are allowed unless individual contraindications exist; aerobic activities are preferred because anaerobic activities might cause too much strain on the skeleton (grade D, weak recommendation)
- We support guidelines for the prevention and treatment of obesity as in the general population (grade D, weak recommendation)

## Potential new treatments

In addition to antibodies directed against FGF23, other drugs aiming at inhibiting the effects of high FGF23 levels are under development, some of which might enter clinical use in future years. For example, the FGF23 receptor antagonist NVP-BGJ398 is in an advanced phase of development^190^. A number of other potential pharmacological targets have also been identified, including antagonizing peptides known to inhibit bone mineralization and regulation of FGF23 protein expression^190–192^.

## Conclusions and perspectives

XLH is a rare chronic disease that substantially alters the quality of life of affected patients throughout life. Knowledge of this condition is unfortunately often restricted to a few specialized centres. This multisystem disease evolves over time, and multidisciplinary care of patients with XLH is needed, involving physicians, physiotherapists, dentists and social workers and liaison with patient group representatives. In these recommendations, we have attempted to cover most features of the disease in order to support such lifelong multidisciplinary care of patients with XLH. Our aim was to identify the criteria for diagnosis, provide guidance for medical and surgical treatment and explain the challenges of follow-up. These recommendations will be updated in the future, in particular when more information on the natural history of the disease becomes available and further data on burosumab emerge. Various topics for future research are outlined in Box 14.

Box 14 Future research topics

- Develop a comprehensive registry for children and adults with X-linked hypophosphataemia (XLH) to evaluate the natural history of the disease, including rare complications
- Evaluate the impact of XLH on schooling, social life and professional activity
- Develop clinical and/or biological and/or radiological scores to support the evaluation of treatment efficacy and safety
- Define the degree of skeletal deformity that is compatible with good quality of life
- Define the risk versus benefit ratio for surgical interventions (osteotomy versus guided techniques)
- Evaluate the risk versus benefit ratio of conventional treatment in adults before and after surgical interventions
- Evaluate the risk versus benefit ratio of conventional treatment in pregnant and lactating women with XLH
- Evaluate the optimal target range of biochemical surrogates (such as fasting serum phosphate levels and maximum tubular reabsorption of phosphate per glomerular filtration rate) in patients on burosumab therapy
- Evaluate the efficacy and safety of burosumab in infants and adolescent patients
- Evaluate the long-term efficacy and safety of burosumab in patients with XLH with respect to critical outcomes such as growth, bone shape, physical function, tooth mineralization, hearing function, neurosurgical complications and prevention of pseudofractures, enthesopathies, tooth abscess and osteoarthritis
- Define the patients who will benefit most from burosumab treatment and should therefore be preferably started or switched to this treatment
- Define the optimal dose and frequency of burosumab treatment to use once patients have achieved a stable disease state
- Evaluate the cost-effectiveness of burosumab and conventional treatment in paediatric and adult patients with XLH

## Glossary

Osteomalacia: Mineralization defect of bone (soft bone) resulting in bone pain and deformations.
Odontomalacia: Mineralization defect of odontoblasts resulting in soft teeth.
Pseudofractures: Atraumatic lucencies (pale area revealed in radiography) extending across one cortex; by contrast, fractures are defined as lucencies extending across both cortices.
Varus deformity: The distal part of the leg is deviated inwards, in relation to the femur, resulting in a bow-legged appearance.
Valgus deformity: The distal part of the leg below the knee is deviated outwards, in relation to the femur, resulting in a knock-kneed appearance.
Dolichocephaly: A condition in which the head is longer than would be expected relative to its width.
Rachitic rosary: The prominent knobs of bone at the costochondral joints in patients with rickets (also known as beading of the ribs); the knobs create the appearance of large beads under the skin of the rib cage, hence the name by analogy with the beads of a Catholic Christian rosary.
Harrison’s groove: A horizontal groove along the lower border of the thorax corresponding to the costal insertion of the diaphragm (also known as Harrison’s sulcus); it appears in rickets because of defective mineralization of the bone — the diaphragm, which is always in tension, pulls the softened bone inward.
Intercondylar and/or intermalleolar distance: The distance between knees (intercondylar) and ankles (intermalleolar). It is used to assess the severity of valgus or varus leg deformities. However, it varies with age and cannot replace regular orthopaedic assessment owing to the high complexity of leg deformities (for example, torsion of limbs) in many patients.
Craniosynostosis: A condition in which one or more of the fibrous sutures in a very young skull prematurely fuses by turning into bone (ossification); because the skull cannot expand perpendicular to the fused suture, it compensates by growing more in the direction parallel to the closed sutures.
Osteotomies: Surgical procedures in which the bone is cut, the two ends realigned to improve shape and/or length and stabilized with internal or external fixation.
Mechanical axis: A line drawn from the centre of the femoral head to the centre of the ankle joint; in a patient who is standing, this line should pass through the centre of the knee. When it does not do so, the mechanical axis is deviated.
Retrognathism: A type of malocclusion that refers to an abnormal posterior positioning of the maxilla (the upper fixed bone of the jaw).
